# Conversational AI should fill the white space in mental health care, not replace humans

**DOI:** 10.1038/s41746-026-03088-1

**Published:** 2026-08-05

**Authors:** Nick Kabrel, Elizabeth C. Stade, Shannon Wiltsey Stirman, Jaan Aru, Johannes C. Eichstaedt

**Affiliations:** 1 Department of Psychology, University of Zurich, Zurich, Switzerland; 2 Digital Society Initiative, University of Zurich, Zurich, Switzerland; 3 Department of Psychiatry and Behavioral Sciences, Stanford University, Stanford, CA USA; 4 Institute for Human-Centered Artificial Intelligence, Stanford University, Stanford, CA USA; 5 Dissemination and Training Division, National Center for PTSD, Palo Alto, CA USA; 6 Institute of Computer Science, University of Tartu, Tartu, Estonia; 7 Department of Psychology, Stanford University, Stanford, CA USA; 8 Decision Sciences, INSEAD, Fontainebleau, France

**Keywords:** Business and industry, Health care, Scientific community, Social sciences

## Abstract

Conversational AI (CAI) is being rapidly deployed in mental healthcare, with much of the focus on chatbots delivering therapy directly to patients. However, this focus overlooks broader opportunities to improve mental healthcare as a system. Rather than replacing clinicians in psychotherapy, CAI may have greater near-term value in parts of the care system where support is limited or absent. We highlight opportunities for CAI in prevention and screening, while on waitlists, between therapy sessions, and after treatment, and identify candidate mechanisms through which CAI could improve care at each stage.

## Introduction

Conversational Artificial Intelligence (CAI) has been increasingly applied in mental healthcare settings since its advent in November 2022, when the public launch of ChatGPT (based on GPT-3.5) dramatically expanded awareness and adoption of large language model-based tools. The dominant vision among technologists has been to build chatbots that mimic therapists, often envisioning a future in which CAI can fully substitute for human clinicians^1,2^. Several randomized controlled trials (RCTs) have demonstrated the efficacy of elements of psychotherapy delivered by CAI-based chatbots^3–5^. For example, a recent first-of-its-kind RCT of a fully generative chatbot showed significant improvements in symptoms of depression and anxiety^6^. These results are preliminary but promising, with perhaps the largest question being whether user engagement can be sustained long enough for meaningful engagement of long-term therapeutic mechanisms^7–9^.

However, we argue that this focus may miss some of the most promising opportunities for CAI to transform the mental healthcare system as a whole rather than replace human clinicians. Like the internet, CAI is a general technological affordance whose impact on healthcare will depend on how thoughtfully it is woven into the broader infrastructure of care. People are already using it to seek mental health support^10^, demonstrating that it will increasingly be seen as a “first responder” and an opportunity to connect individuals with appropriate levels of care.

We argue that CAI should be deployed where it has the greatest relative potential to improve the status quo, following a recent saying in the tech industry that “AI is not better than the best human, but often better than the best available human.” In this case, there are many stages of the mental health care pathway where, currently, “the best available human” is nobody. Increasingly, AI systems appear to be replacing internet search engines and forums as the initial source of information and support for individuals with mental health distress, and thus, they present an opportunity for preventive intervention, or the first point in a stepped care approach, reaching individuals who may never access traditional care.

In most systems, universal screening and prevention efforts are often insufficiently implemented, resulting in undetected cases and unsupported patients, despite the demonstrated value of screening^11,12^. Once a referral is made, patients often face long waitlists. For instance, in the U.S., wait times commonly range from 2–3 weeks to several months^13,14^, with an average delay of around seven weeks, with millions awaiting care^15^. Moreover, therapy sessions are often spaced two to three weeks apart due to insufficient capacity, although research indicates that greater session frequency is associated with better treatment outcomes^16^. Between-session support is typically minimal or inconsistent, compounding the gap in care^17^. Likewise, when patients complete psychotherapy, relapse prevention, and aftercare programs are often minimal^18^, even though the risk of relapse is high, particularly for those with chronic conditions^19^.

### Considering the entire mental healthcare pathway

Experts have long emphasized that a focus on the patient in isolation is insufficient to reduce the overall population burden of mental disorders, pointing instead to systems-level interventions such as universal screening and prevention^20,21^. In these overlooked spaces, even modest AI-based interventions could produce meaningful benefits. We argue that they are a promising target for CAI development efforts in the near term.

**Fig. 1:**
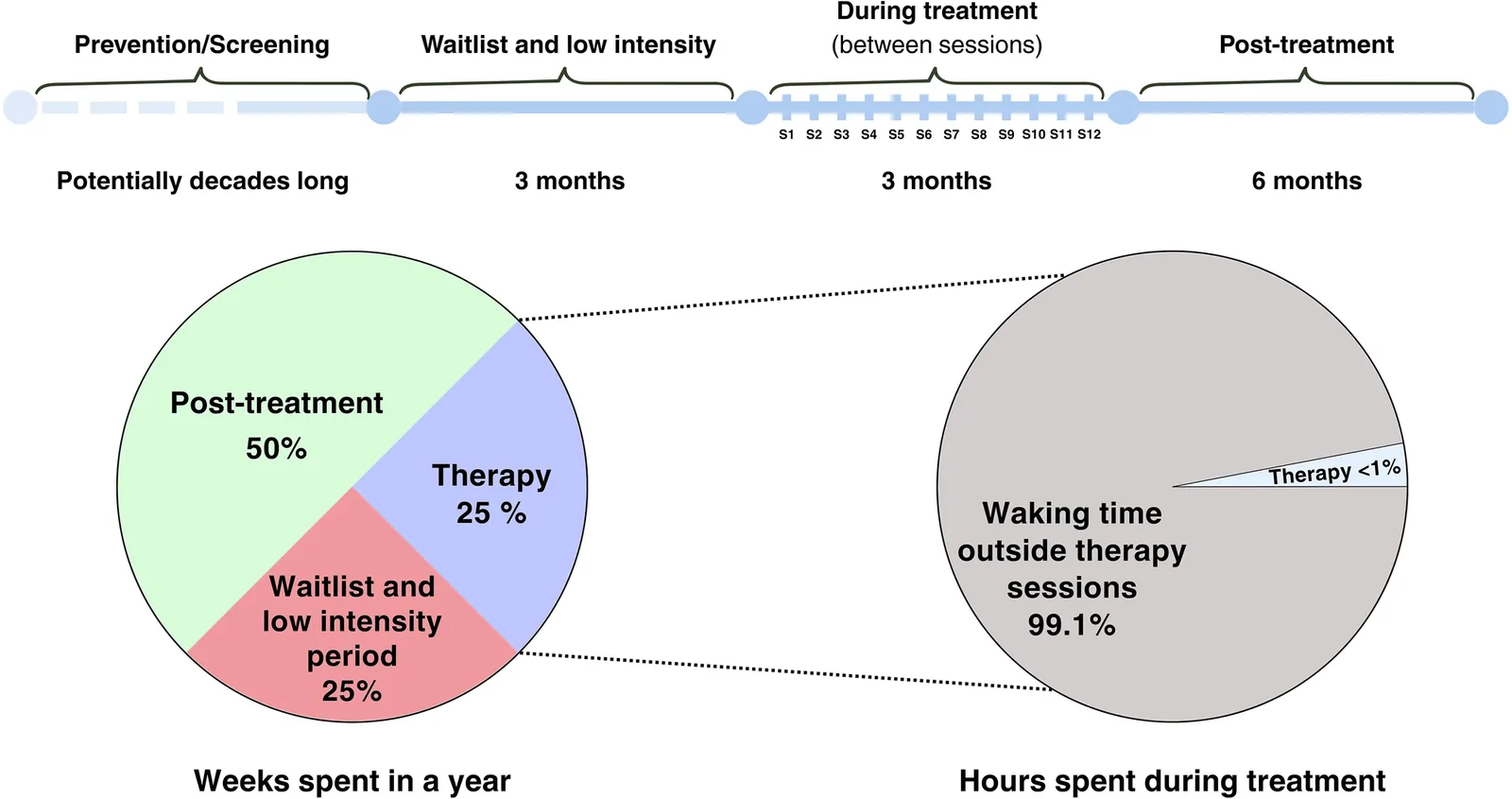
Illustrative proportion of time spent on a mental healthcare pathway over one year. This figure represents an illustrative, typical one-year timeline of the mental healthcare pathway, highlighting the relative proportions of time spent in each stage. Psychotherapy itself typically occupies only a small fraction of the overall process, with much more time spent while waiting for treatment, between sessions, and in aftercare. We relied on published estimates to estimate relative time use: 3 months (≈ 25% of the year) for the waitlist period (based on European healthcare data showing average wait times of 2–5 months; 3 months of weekly psychotherapy (≈ 25% of weeks in a year)^110^ and 6 months (≈ 50%) allocated to aftercare (follow-up and maintenance programs)^111^. For the right pie chart during treatment, we assumed 1 hour of weekly psychotherapy and 16 hours of waking activity per day (and thus 112 waking hours per week).

Although psychotherapy is often seen as central to care, meaningful change can begin well before treatment starts and must often be sustained long after it ends (see Fig. 1). Indeed, empirical evidence shows that mental health disorders can be prevented before they emerge^21,22^ and that aftercare programs help reduce relapses after release from treatment^23–25^.

**Fig. 2:**
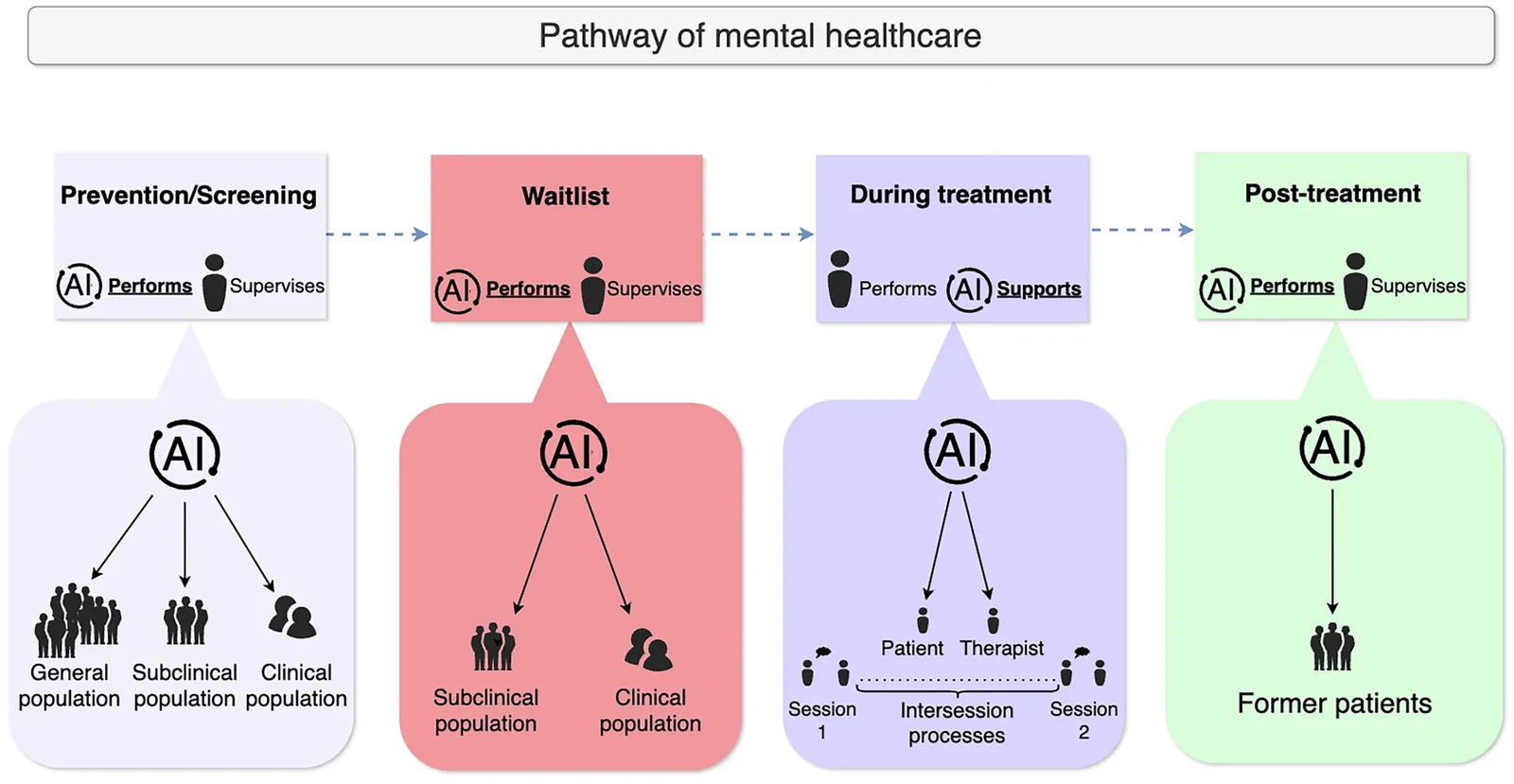
Pathway of care augmented by CAI. There are at least four states in mental health care that can be supported by CAI, either with CAI performing a task under human supervision or a human performing a task with CAI support^112^. The prevention stage can target three distinct populations: the general population, individuals with subclinical conditions, and patients with diagnosed clinical conditions. In the screening stage, individuals exhibiting clinical symptoms are evaluated and may be placed on a waitlist. During the waitlist period, patients may receive support in preparation for psychotherapy, or CAI may conduct symptom assessment/intake. During the treatment stage, both the patient and therapist may be supported between therapy sessions. Finally, the post-treatment stage provides patients with ongoing support to maintain therapeutic outcomes, reinforce acquired skills, monitor for potential relapse, and facilitate timely readmission, if necessary.

Therefore, we propose a “figure-ground reversal”: to focus on using CAI in mental healthcare to support mental healthcare pathways *outside* of psychotherapy. In this paper, we offer four entry points for integrating CAI into the mental healthcare pathway: (1) for prevention—before there is an explicit need for therapy, (2) waitlist—when there is a need for psychotherapy but psychotherapy has not begun, (3) intersession processes—between therapy sessions when therapy is ongoing, and (4) sustainment—after formal psychotherapy has concluded (see Fig. 2).

As we show below, the pathways described in this paper share a common design principle: a trained human remains 'in the loop'. This does not mean, however, that a therapist is present in or monitors every CAI interaction in real time. Rather, it means that a qualified clinician or care coordinator retains responsibility for the patient’s care, that CAI outputs are structured to be reviewable and actionable, and that no clinically consequential decision is made by CAI alone. The precise form of this oversight varies across stages, depending on the clinical context and the stakes involved. For example, at the lighter end, a care coordinator may briefly review an AI-flagged report after a screening interaction and decide whether to escalate the patient to a therapist, which is a low-burden, episodic task with a clear decision point. In contrast, in the therapy stage, a therapist might review a structured CAI summary of intersession activity before each session, providing a more continuous, clinically integrated form of oversight in which the therapist actively incorporates CAI-generated insights into their therapeutic planning.

However, several of the oversight functions we describe, particularly those assigned to care coordinators and intake staff, may add to the responsibilities of roles that in many systems are already overstretched, poorly compensated, and subject to high turnover. Shifting routine monitoring onto CAI relieves the system only if the human review remains sufficiently resourced—otherwise, the bottleneck migrates from clinicians to coordinators. We imagine, however, that the redesign of healthcare systems through CAI may also create a new range of health professionals who are supported by AI to supervise clinical AI systems (“human on the loop,” in the tech world). How these roles are designed, staffed, and trained will require careful thought and investment.

We also acknowledge limits to the scope of the “human in the loop” design principle. We are referring primarily to the care relationship: a clinician or care coordinator (or new kind of professional) remains responsible for the patient and for any clinically consequential decision. We do not mean that every validated CAI tool must be operated under direct clinical supervision. A distinct and already established paradigm, that of prescription digital therapeutics, demonstrates that autonomous, fixed-protocol tools can be deployed as validated medical devices. To illustrate, Rejoyn, a prescription digital therapeutic cleared for major depressive disorder symptoms^26^, and reSET-O^27^, cleared for opioid use disorder symptoms, along with several applications from the German DiGa, are prescribed by a clinician and then used by the patient largely without clinical monitoring, with established safety. We do not argue that human-free tools should not exist, nor that clinician oversight is the only path to patient safety. It is that the open-ended, generative CAI applications we describe in the near term are best deployed within a clinician-managed care relationship. The two models are complementary: where fully autonomous digital therapeutics can already be safely deployed, they can reduce the burden on the healthcare system, while human-in-the-loop CAI extends support into the prevention, waitlist, intersession, and aftercare stages where standalone systems are not yet validated as safe and effective.

Across all stages, a baseline set of safety requirements remains constant: CAI systems must be capable of recognizing indicators of acute risk and responding with immediate safety messaging, provision of crisis resources, and automated escalation to a designated clinical contact, for example, through continuous monitoring of validated symptom measures or detection of language patterns associated with high-risk conditions. While some emerging guidelines are beginning to address these safety requirements^28–32^, comprehensive and empirically validated protocols have yet to be developed, which may become an important priority.

### Prevention

There are three types of prevention models: (1) universal prevention, which is aimed at targeting the overall population irrespective of the risk factors, (2) selective prevention, which targets subpopulations at risk of developing a disorder based on bio-psycho-social risk factors, and (3) indicated prevention, which targets individuals with subthreshold symptoms of a mental disorder who do not yet qualify for a diagnosis^33,34^.

Despite being effective and cost-effective^22,35,36^, mental health prevention strategies do not receive sufficient governmental funding, as they require long periods of engagement and are costly^37^. Using CAI for prevention is promising since it can address some of these limitations. First, CAI technology can be implemented on all three levels of prevention classification: it can target the general population as well as (sub)clinical populations (Fig. 2).

To illustrate, for universal prevention targeting the general population, CAI can be embedded into existing digital ecosystems, such as public health apps, workplace wellness platforms, or educational settings, as optional, user-initiated tools offering psychoeducation, stress-reduction practices, or mood tracking. CAI can be offered as part of routine screening in primary care, for example, during annual check-ups or wellness consultations where patients already expect health-related discussions. For selective and indicated prevention, CAI can support individuals at elevated risk or with subclinical symptoms through tailored interventions, such as through integration with electronic health systems or referrals from school counselors or general practitioners.

Such a strategy is relatively affordable since it does not require intensive human involvement. Furthermore, studies have shown that digital prevention programs administered through web applications and websites have a small but significant effect in preventing mental disorders^36^. Since it is known that more engaging web-based programs lead to better effectiveness^38^, CAI warrants investigation for prevention as it may enhance adherence through greater flexibility and personalization, and thus, engagement^6^.

Extensive research has identified numerous risk and protective factors associated with the development of mental disorders^19,39,40^. CAI could help individuals strengthen protective factors (e.g., emotion regulation or social connectedness) and detect early signs of risk. Such tools could also empower individuals to activate existing psychological and social resources, such as reaching out to trusted peers or engaging in meaningful activities, thereby aligning with a strength-based approach to mental health prevention^41^.

Effective psychoeducation alone might help increase awareness about mental health, address common biases, and reduce stigma. The psychoeducation skills of ChatGPT have been recently evaluated by psychotherapists and found to be adequate^42^.

However, these opportunities must be considered alongside an important contextual challenge: general-purpose LLM-based chatbots (like ChatGPT or Claude) are already widely used for mental health purposes^10,43,44^. These tools are highly accessible, free, and perceived as empathic and non-stigmatizing^45^, making them attractive to individuals who might not otherwise seek support. Yet they operate without clinician oversight, follow no validated clinical protocols, and exist entirely outside any prescribed care pathway. There is a legitimate concern that introducing sanctioned CAI tools could inadvertently normalize broader chatbot use, further substituting human therapeutic engagement. Yet this risk works both ways: unregulated general-purpose chatbot use for mental health is already growing regardless of what happens in clinical care. Perhaps a more productive question is whether a clinician-supervised, validated CAI tool embedded in a care pathway is persuasive to users as a superior option to general-purpose chatbots. This also means that the patients need to hear and understand why they should prefer these clinically validated chatbots to standard chatbots, and that the clinical alternatives cannot be notably less accessible and harder to use than ChatGPT. Clinicians could actively guide patients toward validated tools during consultations, while public health authorities could play a role in raising broader awareness.

It is also important to distinguish between prevention applications that are immediately deployable and those requiring more extensive regulatory work. Universal prevention tools, offering psychoeducation, stress management, or general well-being support, do not make clinical determinations and are unlikely to fall under medical device regulation in most jurisdictions, making them genuinely near-term opportunities. Selective prevention, by contrast, may involve identifying at-risk or subclinical individuals, which, as a clinical assessment, could require evaluation as Software as a Medical Device (SaMD) under frameworks such as the FDA’s or the EU Medical Device Regulation (MDR). Future development efforts should be explicit about where on this spectrum a given tool sits in order to both set realistic deployment timelines and to realistically anticipate barriers to implementation.

Hence, future research should systematically investigate how CAI can be most effectively tailored and deployed across the different levels of prevention. This includes identifying which conversational strategies, content formats, and delivery contexts are most beneficial for diverse populations. Controlled trials comparing CAI-based interventions with existing digital and human-delivered preventive approaches are needed to evaluate their effectiveness, adherence, and long-term impact.

### Screening

Prior work has found that general practitioners do not have the time or resources to conduct even short routine screenings for mental disorders^46,47^. CAI could support assessment, for example, by engaging patients in short conversations at the beginning or end of a medical visit to flag potential mental health concerns. This could both enhance prevention and reduce the burden on already overloaded general practitioners. Similarly, a brief, structured check-in with a chatbot during school or workplace health assessments could deliver programs that reduce risk factors for anxiety and depression^48^.

Although it is perhaps too early to speak about CAI diagnosing a condition and severity in real-world settings, some studies suggest that it may become a feasible option in the future^49^. For example, studies show that chatbots might be able to detect mental issues by asking sets of questions based on screening questionnaires, similar to clinicians^49–52^.

Additionally, Rollwage et al.^53^ recently tested a CAI-based self-referral tool. This conversational chatbot is integrated into the service’s website and assists patients by collecting the necessary intake information (eligibility criteria, demographic information, etc). This additional information includes free-text input regarding the patient’s presenting symptoms as well as standardized, clinically validated routine outcome measures and screening questions. The outcomes of this program showed that the use of CAI increased clinical efficiency by reducing the time clinicians spend on preliminary mental health assessments and reducing wait times for patients, reducing dropout rates, and increasing recovery rates^53^. The study found the sharing of clinically relevant information obtained through CAI with service providers to be particularly important.

In this context, human-in-the-loop oversight takes the form of a care coordinator or GP receiving a report that has been flagged by AI and retaining full responsibility for any subsequent clinical decision, i.e., CAI flags concerns, but does not decide escalation alone.

Research should explore further how to integrate CAI ethically and equitably into real-world care infrastructures—such as schools, workplaces, or primary care settings—without overwhelming users or providers.

### Waitlist periods and low intensity care

Long waitlist time is a formidable challenge for mental healthcare systems across the world^54^. For instance, the general waitlist time for psychotherapy is up to 3 months in the US^13,14^ and ~4–5 months in Germany^55^. There’s emerging acceptance of the idea that waitlists are harmful to people’s mental health: in general, the condition of prospective therapy patients deteriorates while being on a waitlist^56–59^. Building on previous developments in computerized CBT programs^60^, using CAI-based tools to support individuals during the wait for treatment represents a promising and scalable solution during a time when they would otherwise see deterioration in their symptoms^61,62^.

Another important aspect is that many patients require a substantial number of therapy sessions before experiencing meaningful improvement. Given that 9–13 sessions can be needed for meaningful clinical change^63,64^, one promising strategy to reduce this delay is to offer low-intensity interventions prior to therapy. In fact, programs such as the United Kingdom’s NHS Talking Therapies program offer low-intensity, often digital interventions to eligible patients as part of a stepped-care model before offering more frequent therapy^61^. There is also emerging evidence that therapists trained in both low- and high-intensity approaches can deliver these flexibly based on patient need, resulting in fewer sessions and faster recovery compared to the standard sequential stepped-care pathway^65^. CAI could naturally supplement and enhance such stepped-care services, providing a personalized and continuously available complement guided self-help and internet-delivered CBT. Additionally, single-session interventions have been shown to produce measurable improvements for individuals awaiting treatment^48^. Likewise, single-session consultations (SSC), which prompt patients to identify and implement achievable behavior changes while on waitlists, have demonstrated feasibility and symptom reduction in both in-person and telehealth formats^66,67^.

In addition, there is strong evidence that role induction—providing patients with information about what to expect from therapy and how to engage effectively—improves early engagement and outcomes^68^. Similarly, studies indicate that motivational interviewing (MI) before treatment can enhance retention and outcomes^69,70^. Together, these findings suggest that evidence-based pre-therapy interventions can accelerate therapeutic gains and reduce dropout. CAI could provide a scalable way to deliver such interventions, improving readiness for therapy while helping to alleviate systemic bottlenecks in access to care.

Such an approach can also account for a practical human oversight during the waitlist or low-intensity period. While no individual therapist may yet be assigned, the CAI tool could operate within the service’s infrastructure, with intake coordinators receiving automated alerts if symptom scores or risk indicators cross predefined thresholds, ensuring that deterioration is addressed. CAI-generated intake summaries and symptom profiles could then be shared with the assigned therapist before the first session, informing their clinical approach without requiring them to review raw interaction logs, which would unnecessarily increase workload.

Based on this section, future proof-of-concept studies could test whether chatbot interventions can activate specific psychological mechanisms known to support treatment outcomes. For instance, a brief chatbot conversation conducted before the first therapy session might increase a patient’s positive expectations toward treatment, which is a well-established predictor of therapeutic success^71^. In medicine, proof-of-concept studies demonstrate that an intervention engages its intended mechanism (e.g., a drug reaches the bloodstream or alters a biomarker)^72^. Similarly, in mental health, we need preliminary studies to assess whether key change mechanisms can be effectively targeted by digital tools before therapy.

### Between therapy sessions

Psychotherapy sessions typically occur once a week for about one hour, which is a tiny fraction of the waking or working hours in a given week (Fig. 1). While much attention has been given to in-session factors that contribute to change^73^, the time between sessions also plays a significant role^17^. Assuming the person is awake for 112 hours per week (based on 16 waking hours per day), 99%+ of those hours are spent in between sessions. Intersession processes^17^ are important and include patients’ reflections on the previous sessions, their internalized image of psychotherapy, and thoughts about their therapist, along with different homework assignments. Since these processes occur in patients’ daily lives and it is challenging to track them, supporting these processes with CAI may be a fruitful target.

Specifically, CAI can help patients practice and consolidate skills learned during therapy—a powerful mediator of lasting change^74–76^. Meta-analytic evidence indicates that both the quantity and quality of homework compliance predict therapy success across cognitive-behavioral interventions^77,78^. Despite this potential, much of the time between sessions is underutilized: patients often do not fully engage with exercises or apply skills in daily life. CAI-based applications could fill this gap by guiding patients through homework assignments, providing reminders, answering questions about exercises, or helping interpret experiences (e.g., distinguishing thoughts from emotions), thereby reinforcing skill acquisition and supporting behavioral change^28,79^. Supporting this view, a recent real-world study found that patients using an AI-enabled therapy support tool alongside group CBT demonstrated higher session attendance, lower dropout, and greater clinical improvement compared to those using standard workbooks, with benefits linked to engagement and personalization^80^. Similarly, Sharma et al.^81^ have found that self-guided LLM-based exercises might facilitate adaptive cognitive restructuring.

Beyond homework support, CAI may also facilitate additional processes such as reflection and insight generation. For example, chatbots can help organize patient reflections, summarize intersession experiences, or track cognitive and emotional patterns, which could then be summarized by CAI and reviewed by the therapist in subsequent sessions. Multi-modal CAI systems with increasingly seamless speech-to-text capabilities can make these processes more convenient, allowing patients to log experiences as voice notes^82^. Summarization is one of the strongest use cases for CAI^61^; thus, daily or weekly summaries with key points could be created based on such recordings, which patients can share with their therapist. Also, these systems can extract the main cognitive patterns, e.g., patients’ negative thoughts and certain emotional reactions, and illuminate the connections between them, for which self-insight might be limited.

In general, the use of digital tools to complement face-to-face therapy has been studied under the broader umbrella of “blended care,” i.e., interventions that purposefully combine in-person and internet- or app-based components^83^. Systematic reviews suggest that blended approaches are feasible and generally more effective than no-treatment controls, and in some studies may offer advantages over stand-alone face-to-face therapy, including lower dropout rates and improved maintenance of treatment gains^84^. A more recent systematic review and meta-analysis by Nunes-Zlotkowski et al.^85^ found evidence supporting the effectiveness of blended therapy (particularly for depression). Taken together, this body of work provides an important precedent for CAI-augmented between-session support.

Future research should examine how CAI can most effectively support intersession processes. A key practical question concerns the feasibility of therapist oversight within existing clinical workloads: rather than reviewing full transcripts (which would be neither feasible nor necessary), therapists could engage with CAI-generated weekly summaries of intersession interactions, structured to highlight key themes, mood trends, and homework engagement. This use case plays to efficient summarization as one of the core strengths of CAI. Crucially, this model means CAI actually reduces rather than adds to therapist burden, since the relevant information is available pre-processed at the start of each session rather than having to be elicited within the session. As therapists become more familiar with this workflow, one could also imagine that they specifically prompt CAI to look out for themes they deem to be of particular therapeutic relevance at that moment. Studies could explore which summary formats, levels of detail, and foregrounding of clinical mechanisms are most useful, and how therapists can incorporate this information into their clinical decision-making. Ideally, such solutions would co-evolve with therapist preferences and workflows in user-centered design processes^86,87^. Last but not least, longitudinal trials are needed to assess whether CAI-enhanced intersession engagement leads to better therapeutic outcomes.

### Post-treatment

Mental disorders are, in general, highly recurrent and are prone to relapse and chronification^19,88,89^, with depression being a prime example, characterized by a particularly high recurrence^19^. While it is known that evidence-based psychotherapy is generally effective^90,91^, these effects often tend to decrease after psychotherapy is finished^92^. Hence, follow-up aftercare programs, including outpatient psychotherapy and monitoring, are recommended for people with depressive disorders^23^. The main aim of this period is to consolidate treatment outcomes and ensure their longevity^18^.

Studies show that aftercare can reduce rates of relapse, as well as increase rates of appropriate treatment following relapse due to increased monitoring^93,94^. This might allow clinicians to reduce the risk of relapse. However, one of the main barriers to administering effective aftercare programs is the limited resources of healthcare systems, given high costs^18^. To address these issues, in recent years, innovative low-cost and low-threshold aftercare models have emerged, such as web- or telephone-based follow-up monitoring programs^95–97^.

Building on these developments, CAI could offer structured, interactive modules tailored to post-therapy needs. For example, a CAI system could guide users through periodic relapse monitoring using validated mood and symptom questionnaires, prompting reflection on triggers and early warning signs, and offering personalized relapse prevention, prompting the patient to make use of the skills that worked the best for them in therapy. It could also provide personalized psychoeducation refreshers, facilitate structured behavioral activation exercises, and offer motivational dialogue to sustain therapeutic habits. Importantly, the CAI system could escalate cases by notifying clinicians when risk thresholds are exceeded.

Similarly to the concern mentioned while discussing prevention opportunities, not all of these applications are equally immediate. Coaching-style functions, reminding users to engage with their relapse prevention plan, prompting reflection on early warning signs, or encouraging clinician contact, are unlikely to require medical device approval in most jurisdictions. These represent genuinely near-term opportunities, deployable within existing care frameworks. More personalized functions, for example, adapting interventions to evolving symptom profiles, delivering tailored clinical content, or autonomously escalating risk, involve clinical judgment and would more plausibly require Software-as-Medical-Device approval before deployment.

In addition, the post-treatment phase warrants specific attention regarding human oversight, since patients may have been formally discharged and no longer have an assigned treating therapist. In this context, a practical oversight model could involve CAI conducting brief periodic check-ins that combine validated symptom measures with open-ended conversational assessment, therefore capturing not just scores but the patient’s own account of their current state, emerging stressors, and engagement with their relapse prevention plan. CAI could then generate a concise, structured summary for the patient’s care coordinator, flagging deterioration risk. This goes meaningfully beyond what a static questionnaire can offer, while keeping clinical decision-making with the patient and human reviewer. Longer term, developing standardized CAI protocols capable of reliably detecting acute risk in post-treatment contexts would be valuable, and published findings already suggest that this is feasible in the near term. For example, Swaminathan et al.^98^ have already demonstrated high sensitivity for detecting crisis signals in clinical chat messages in telehealth, and Guo et al.^99^ have found that an LLM-based PHQ-9 screening chatbot showed strong agreement with the surveys and high user acceptability in a multi-country evaluation^99^.

That said, future studies should investigate the efficacy and acceptability of CAI-based aftercare interventions in sustaining treatment gains and preventing relapse, particularly among individuals with recurrent conditions like depression or psychosis. RCTs could compare CAI-supported aftercare with traditional follow-up models and evaluate outcomes such as relapse rates, user engagement, and cost-effectiveness. Research should also explore which components (e.g., mood tracking, motivational dialogue, or elements of CBT) are most impactful and how to adapt these for varying clinical profiles.

### CAI support for therapists

In addition to filling the gaps for therapy patients, CAI can also assist therapists outside of therapy proper. Based on the transcripts of psychotherapy sessions, CAI can perform a number of actions at different levels of analytic depth. On the basic level, it can summarize the session, find key points discussed in the session, and identify areas for progress, akin to meeting “co-pilots” that are being routinely implemented in video conferencing solutions, like Zoom or Teams. Indeed, one of the obvious opportunities lies in supporting the non-patient-facing administrative tasks that often consume a substantial portion of therapists’ time. CAI systems can generate structured summaries of therapy sessions^100^, thereby reducing the burden of routine documentation. Automatic CAI-based services to deliver such documentation have experienced rapid adoption in other parts of medicine^101^.

That said, the benefits should not be overstated. Evidence from general medicine suggests that while AI-assisted documentation might reduce burnout and increase well-being, demonstrated effects on objective productivity so far are modest^102^. This is partly an integration problem: if reviewing and correcting AI-generated notes consumes significant time, net savings may be minimal, and poorly integrated tools risk losing buy-in from therapists. For CAI documentation support to deliver on its promise, it must fit naturally into clinical workflows rather than merely shifting effort. Additionally, patients have endorsed varying levels of trust and comfort in these systems, and their use when discussing sensitive topics needs to include opt-out options and assurances with respect to how the information is stored and used^103^.

An even subtler concern is the risk of deskilling. Writing session notes is, in part, a reflective clinical practice. When CAI generates this narrative, the therapist shifts from author to receiver, approving an account rather than constructing their own. Preliminary research on cognitive offloading suggests that outsourcing such tasks to AI can reduce the neural engagement associated with memory, pattern recognition, and metacognitive awareness over time^104,105^. The design challenge, then, is to find the right level of assistance. While fully automated notes risk deskilling, writing notes from scratch is a demonstrated burden. More balanced models will have to be developed; for example, CAI could surface key moments from session transcripts as the basis for reflection and case conceptualization.

Beyond session notes, CAI systems can assist in drafting referral requests, preparing statements for insurance providers, and maintaining up-to-date clinical records^101^. By taking on these administrative roles rather than the therapeutic relationship itself, CAI can improve efficiency, reduce clinician cognitive load, and allow therapists to concentrate on core therapeutic tasks and higher-order decision-making^102,106^. Supporting the superordinate administrative structure of healthcare is, therefore, a meaningful avenue for supporting mental health care with CAI.

On a deeper level, CAI that is grounded in particular evidence-based treatment manuals could act as consultants for psychotherapists^28,107^ and generally provide actionable feedback. For instance, it can help with identifying the effective and ineffective aspects of the therapist’s behavior throughout the session. It can also analyze the behavior of the patient in the session, for example: were there some moments when the patient avoided some questions, or the therapist ignored points the patient brought up?

In addition, CAI systems could be used to monitor established mechanisms of change within evidence-based treatments, offering therapists actionable information between sessions. For example, in cognitive therapy for PTSD, changes in trauma-related beliefs are central to recovery and strongly predict treatment outcome^108^. Similarly, in exposure-based interventions, therapeutic progress is often mediated by expectancy violation processes (patients learning that feared outcomes do not occur), which can be assessed and tracked^109^. A CAI-based system could analyze session transcripts or patient reflections to detect such shifts, flagging them for therapist review. The therapist then decides whether and how to address, reinforce, or further explore these mechanisms with the patient. In these applications, CAI can serve as an analytical aid rather than a decision-maker. The interpretation of flagged patterns and the clinical response remain the therapist’s responsibility, preserving judgment and cognitive engagement by the therapist.

Future research should investigate how CAI-based supervision tools can be reliably integrated into clinical training without undermining the development of therapists’ critical thinking, intuition, and interpersonal sensitivity. Empirical studies could explore the effectiveness of CAI feedback in improving specific therapist competencies across different therapeutic modalities and training levels. Additionally, research is needed to determine how therapists interact with, interpret, and act on CAI-generated insights, especially in complex or ambiguous clinical cases.

### Concluding remarks

Since the advent of advanced CAI, the dominant view among technologists has been to use it to directly provide psychotherapy. We argue for a broader perspective on CAI’s role in mental healthcare. Rather than positioning it as a substitute for (human) therapy, CAI can be thought of as a new technological affordance (like the internet) that can be used to transform healthcare systems to make them more integrated, less burdensome, improve their reach, and thus, their impact on public health. To start, the biggest opportunities may lie in areas in which currently little or no human support is available to deliver or support care.

It is also worth acknowledging a more fundamental shift that is already underway. A significant portion of people may never engage with the traditional mental health system. The reason is not only because they cannot access it, but because they choose not to—thanks to the broad availability of general-purpose chatbots that to many appear “good enough.” CAI embedded in healthcare systems offers a way to acknowledge these preferences and reach people who would otherwise remain entirely outside any form of supported care, particularly in prevention and low-intensity support where the potential for population-scale impact is greatest. Rather than waiting for these individuals to enter formal care pathways, the field needs to develop the solutions, regulatory frameworks, evidence, and oversight standards that make such CAI-based support safe and effective, and develop CAI-assisted systems that make human oversight sustainable, and overcome the substantial implementation challenges of changing healthcare systems.

Future research and private sector investment could focus on these gap areas, spanning prevention, waitlist periods, intersession processes, and post-treatment stages. Evaluation of effectiveness would shift from learning a cognitive skill through AI^81^ to the cost-effectiveness and efficiency of the health care system as a whole. To summarize, then, the question is not “Is CAI better at therapy than humans?” but “Will a health care system with integrated CAI be better than a system without?” Based on much preliminary work, we feel the answer is demonstrably yes, and private and academic innovation should “think bigger” about the system as a whole.

## Data Availability

No datasets were generated or analysed during the current study.
